# Prevalence and location of the secondary mesiobuccal canal in 1,100 maxillary molars using cone beam computed tomography

**DOI:** 10.1186/s12880-016-0168-2

**Published:** 2016-12-01

**Authors:** Pablo Betancourt, Pablo Navarro, Gonzalo Muñoz, Ramón Fuentes

**Affiliations:** 1 Research Center in Dental Sciences (CICO), Endodontic Laboratory, Dental School, Universidad de La Frontera, Temuco, Chile; 2 Dental School, Universidad de La Frontera, Temuco, Chile; 3 Integral Adultos Department, Dental School, Universidad de La Frontera, Claro Solar 115, Temuco, Chile

**Keywords:** Maxillary molars, Second mesiobuccal canal, Location, Cone-beam computed tomography

## Abstract

**Background:**

Several articles have used cone beam computed tomography (CBCT) to study the morphology of the maxillary molars and to ascertain its ability to visualize the second mesiobuccal canal (MB2); however, its geometric location has not been examined in depth. The aim of this study was to describe in vivo the prevalence and location of the MB2 in the mesiobuccal root of the first maxillary molar (1MM) and the second maxillary molar (2MM) through CBCT imaging.

**Methods:**

Five hundred fifty CBCT images of the 1MM and 550 of the 2MM were analyzed. To detect the MB2 canal, the observation and measurements were done 1 mm apically to the pulpal floor to standardize the methodology. The geometric location of the central point of the MB2 canal (PMB2) was measured in relation to the central point of the mesiobuccal canal (PMB1) and in relation to the line projected between the PMB1 and the central point of the palatal canals (PP). The data were analyzed using descriptive statistics, with a value of *P* < 0.05 being statistically significant.

**Results:**

In the 1MM, the prevalence of the MB2 canal was 69.82% and was more frequent in women (*p* = 0.005). The distance between PMB1 and PP was 7.64 ± 1.04 mm. The average distance between PMB1 and PMB2 was 2.68 ± 0.49 mm, and for PMB2 and the line projected between the PMB1 and PP canals was 1.25 ± 0.34 mm. In the 2MM, the MB2 canal was identified in 46.91% and was more frequent in men (*p* = 0.000). The distance between PMB1 and PP was 7.02 ± 1.30. The average distance between PMB1 and PMB2 was 2.41 ± 0.64 mm, and for the PMB2 and the line projected between the PMB1 and PP canals was 0.98 ± 0.33 mm.

**Conclusions:**

The MB2 canal was found in a high percentage of the sample. These results indicate that CBCT is an effective, high-precision diagnostic tool not only for detecting but also locating in vivo the MB2 canal in the mesiobuccal root of upper molars.

## Background

The permanent first maxillary molar (1MM) and permanent second maxillary molar (2MM) are the teeth that present the greatest complexity and variation in the root canal system [1, 2], and this is reflected in them having the highest rates of endodontic failure and being a constant challenge for the clinician [3].

**Fig. 1:**
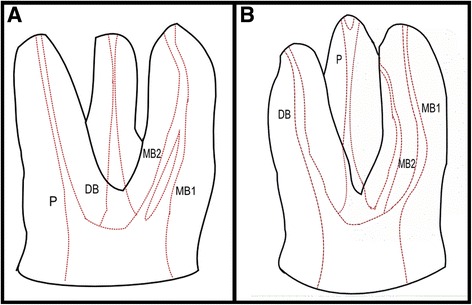
Maxillary molar with 4 canals: first mesiobuccal canal (MB1), secondary mesiobuccal canal (MB2), distobuccal canal (DB) and palatal canal (P). **a** Maxillary molar with joining mesiobuccal canals. **b** Maxillary molar with two separate mesiobuccal canals

A high percentage of treatment failures is due to the impossibility of detecting the presence and location of the secondary mesiobuccal canal (MB2), located in the mesiobuccal root of the 1MM and the 2MM [4], which prevents the correct implementation of biomechanical instrumentation, irrigation and obturation (Fig. 1). Its location in clinical practice is highly complex due to the excessive dentin deposited in the opening of the canal and to the difficulty in visually accessing maxillary molars.

The percentage of visualization of the MB2 canal varies according to the technique used in each study, including histological sections [5], diaphanization [6], magnifying loupes [7], endodontic surgical microscope [8–10], scanning electron microscope [5], micro-computed tomographic analysis [11], and cone beam computed tomography (CBCT) [1, 3, 4, 12].

In recent years, CBCT has made it possible to visualize hard-to-reach anatomical structures in three dimensions, and it has become a valuable aid as a complementary examination for endodontic diagnosis and treatment with a lower dose of radiation than conventional computed tomography [13, 14]. Several articles [1, 3], [4, 12, 15], have used CBCT to study the morphology of the maxillary molars and to ascertain its ability to visualize the MB2 canal; however, its geometric location has not been examined in depth.

The aim of this study was to determine in vivo the prevalence of the MB2 canal in maxillary molars, and to describe a methodology to enable its geometric location through CBCT imaging.

## Methods

This study was approved by the Ethics Committee of the Universidad de La Frontera, Temuco, Chile (Protocol n° 048/13). CBCT images that contained the 1MM and 2MM, from patients both men and women, were analyzed, The images were taken between January 2014 and March 2015, and belong to the radiology unit of the Universidad de La Frontera. The patient’s identity was not revealed and only access to information regarding age and gender was provided.

The imaging examinations were taken as part of the diagnosis, examination and planning of surgical, endodontic, periodontal, orthodontic or rehabilitative treatment. The images were obtained on a Pax Zenith CBCT unit (Vatech, Hwaseong-si, Korea), using 120 kV and 9 mA; FOV 8 × 6 cm, voxel size 0.12 mm.

550 1MM (right and left) and 550 2MM (right and left) CBCT images were included where the presence of all maxillary molars could be observed. Inclusion criteria for the CBCT images were: aged between 15 and 75 years, and complete root formation. The exclusion criteria were: present metallic restoration, intra-radicular post or endodontic filling, rehabilitated using fixed prosthesis, canal calcification, evidence of radectomy or periapical surgery, and maxillary molars with developmental anomalies.

A learning process to reach a consensus in the identification of the MB2 based on the anatomical diagnosis of CBCT images took place prior to a data reliability assessment, because the MB2 is very fine, which reduces the contrast on the image, and its visualization also varies according to the area of the tooth in which the measurement is taken. Two endodontics specialists examined 20 previously selected CBCT images of maxillary molars. The observers analyzed the images on three occasions, at one-week intervals. When a consensus could not be reached, a radiologist with experience in endodontics helped to make the decision. The reliability data were analyzed using the Kappa concordance index, which determined that there was agreement between the observers (*p* = 0.000) and the strength of agreement was very good (0.886).

### Observation methodology

**Fig. 2:**
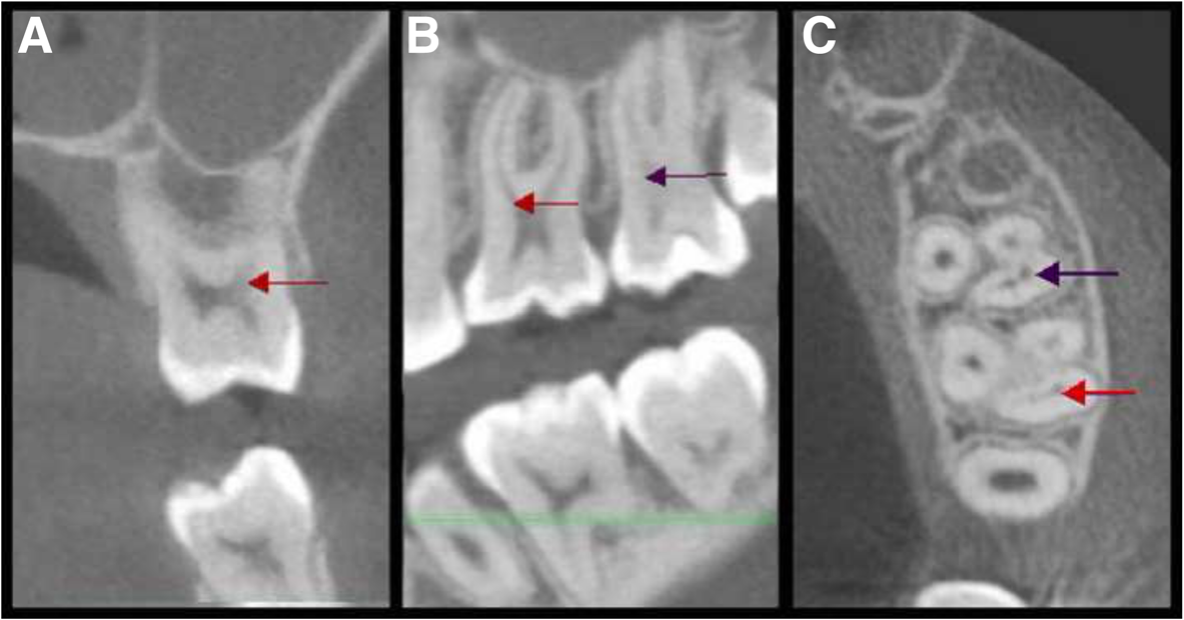
CBCT images of a left maxillary first molar (red arrows) and left maxillary second molar (purple arrows). Mesiobuccal root with 2 canals as viewed in coronal, sagittal, and axial directions using Ez 3D 2009 software. **a** coronal view; **b** sagittal view; **c** axial view

**Fig. 3:**
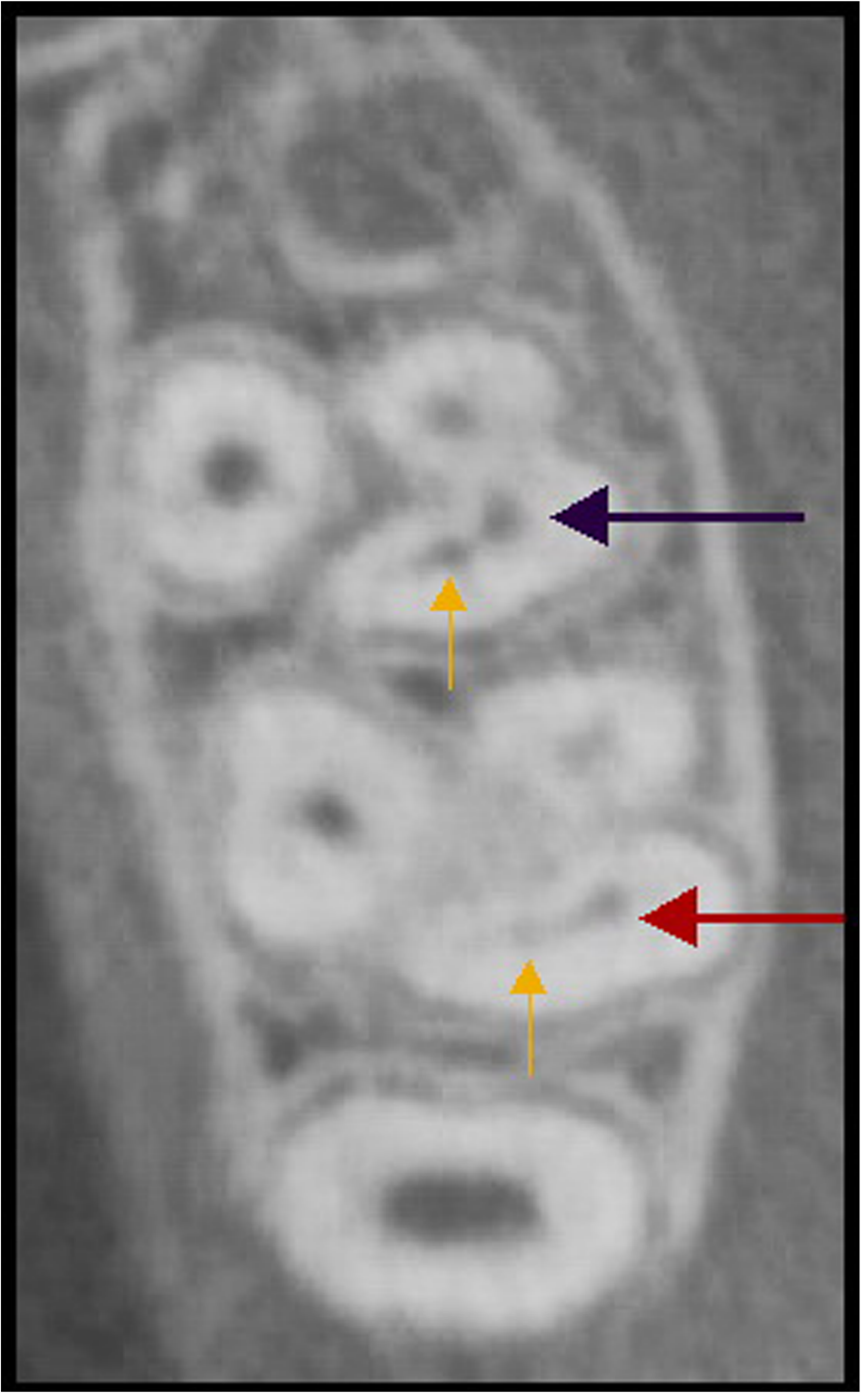
Cross-sectional CBCT image of left maxillary first and second molar with a clearly distinguished MB2 canal (yellow arrows). The red arrows denote the mesiobuccal root in the maxillary first molar and the purple arrows denote the mesiobuccal root in the maxillary second molar

The images were processed with the Ez 3D 2009 software (Vatech, Hwaseong-si, Korea) and projected onto a LED KDL-42W651A screen (Sony, Minato, Japan) to observe coronal (Fig. 2a), sagittal (Fig. 2b) and axial sections (Fig. 2c). First, the sagittal and coronal sections was oriented parallel to the long axis of the root, and then sections were obtained on the axial plane at 0.5 mm intervals and a 1mm thickness for all the samples, using multiplanar reformatting (MPR). MPR constructs a three-dimensional model and shows all structures within the 1mm thickness overlapped on each other. A corono-apical exploration was made. To detect the MB2 canal, the observation and measurements were done 1 mm apically (2 sections of 0.5mm) to the pulpal floor to standardize the methodology (Fig. 3).

**Fig. 4:**
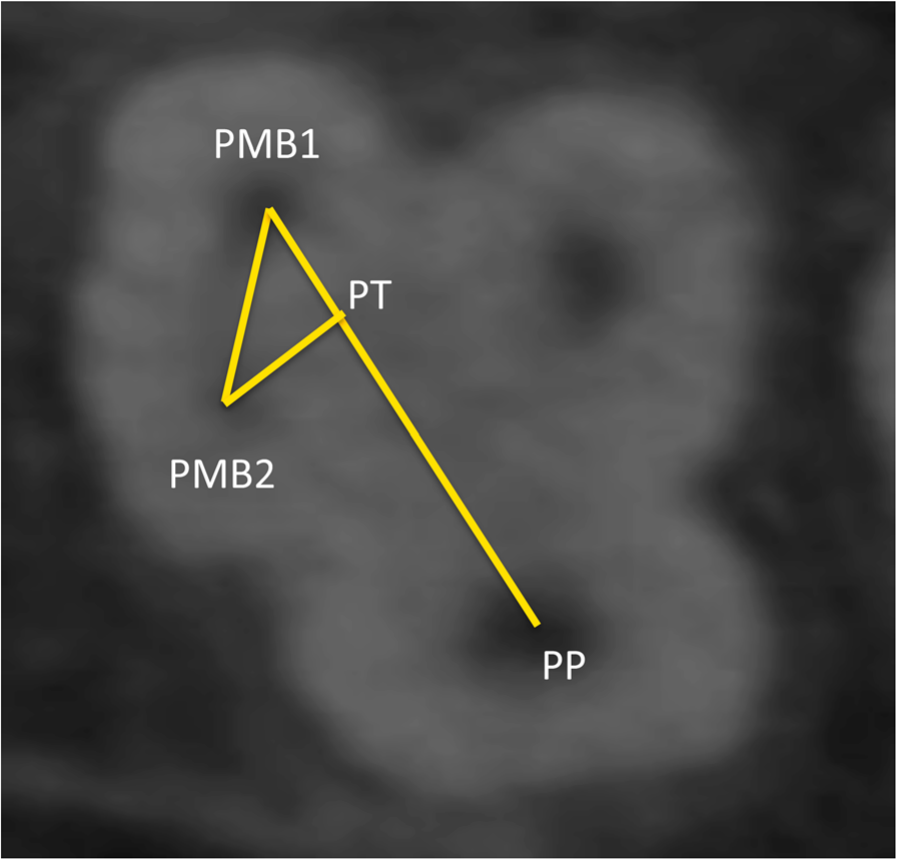
Axial view of left maxillary first molar. PMB1 (center of mesiobuccal canal), PMB2 (center MB2 canal), PP (center palatal canal). Straight lines were projected, joining the different points: PMB1-PP line and PMB1-PMB2 line. A third line was drawn, PMB2-PT, corresponding to a perpendicular line between PMB2 and the PMB1-PP line (PT point). The distance in the lines drawn between the points was measured in millimeters

The geometric location of the MB2 canal was found in relation to the first mesiobuccal canal (MB1) and the palatal canal (P). The central points of each canal were located (PMB1, PMB2 and PP) and straight lines projected between them (PMB1–PP and PMB1–PMB2). A third line was drawn (PMB2–PT), perpendicular to the PMB1–PP line (PT point), according to the protocol described by Betancourt et al. [14]. The distances of the lines drawn between the points were measured in millimeters (Fig. 4).

The data were analyzed using descriptive statistics (mean ± SD). The association between the MB2 canal and gender and side were determined and evaluated using Pearson’s chi-square test with the SPSS/PC v. 20.0 software (SPSS, Chicago, IL). The relation to age was also established using the *t*-test for independent samples, considering the normality of the data. 95% confidence intervals were used to calculate the average distances between the points PMB1-PMB2, PMB1-PP and PMB2-PT. A value of *p* < 0.05 was chosen as the threshold for statistical significance.

## Results

550 1MM and 550 2MM were selected according to the established dates and inclusion criteria.

### First maxillary molar

**Fig. 5:**
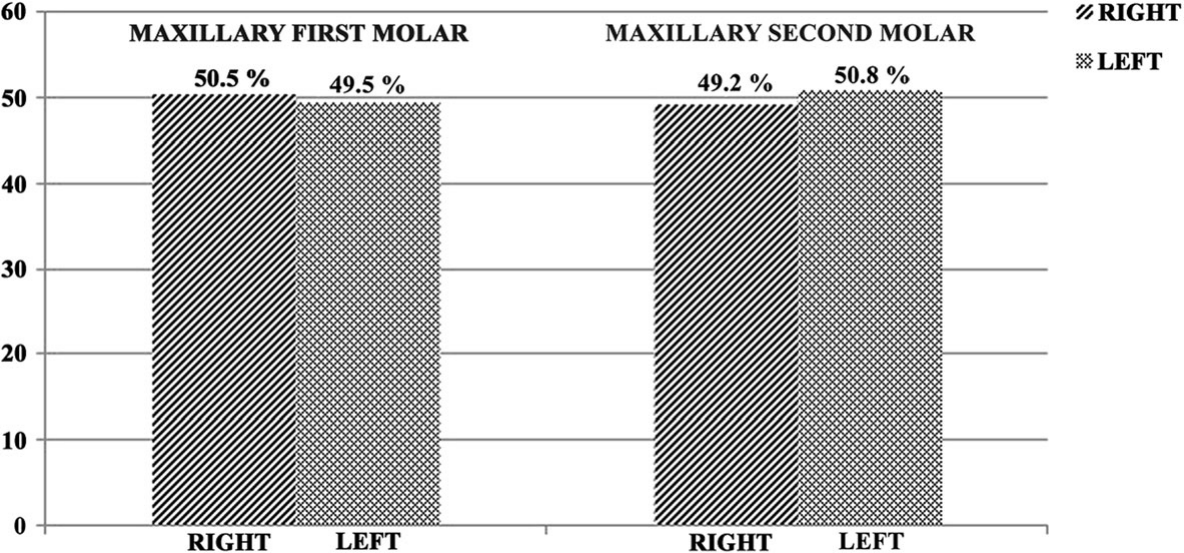
Distribution of the prevalence of the MB2 canal according to side

**Fig. 6:**
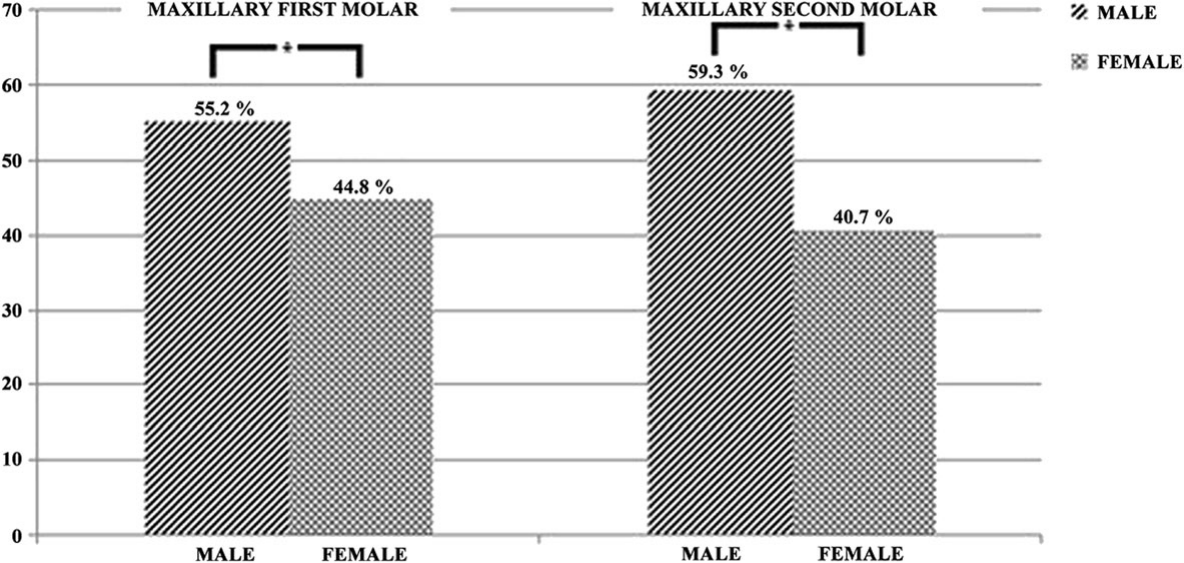
Distribution of the prevalence of the MB2 canal according to gender. *Refers to *P* < 0.05

The MB2 canal was found in 69.82% of the analyzed cases (384/550). The percentage distribution of the MB2 canal according to side was homogenous: 50.5% on the right and 49.5% on the left (Fig. 5). With regard to the incidence of the MB2 canal according to gender, statistically significant differences were observed (*p* = 0.005), with 55.2% in men and 44.8% in woman (Fig. 6) (Table 1). The average age of the subjects where the MB2 canal was found was 27.40 ± 12.95 years. The distances between the points were analyzed with 95% confidence. The distance between PMB1-PP was 7.64 ± 1.04 mm. For PMB1-PMB2 the average of distance was 2.68 ± 0.49 mm, and for PMB2-PT it was 1.25 ± 0.34 mm.

**Table 1:** Prevalence MB2 canal in the mesiobuccal root of the maxillary first molars by gender and tooth position

	MB2		
	Absent	Present	Total
Female	96	172	268
	57,8%	44,8%	48,7%
Male	70	212	282
	42,2%	55,2%*	51,3%
Total	166	384	550
	100%	100%	100%
Rigth Side	85	194	279
	51,2%	50,5%	50,7%
Left Side	81	190	271
	48,8%	49,5%	49,3%
Total	166	384	550
	100%	100%	100%

* refers to *p* < 0.05

### Second maxillary molar

The MB2 canal was identified in 46.91% (258/550) of the cases. When the incidence of the MB2 canal was compared between the right side (49.2%) and left side (50.8%), there were no statistically significant differences (*p* = 0.560) (Fig. 5). Visualization of the MB2 canal was more frequent in men (59.3%) than in women (40.7%), with statistically significant differences between the two genders (*p* = 0.000) (Fig. 6) (Table 2). The average age of the subjects where the MB2 canal was found was 27.81 ± 12.66. The distances between the points were analyzed with 95% confidence. The distance between PMB1-PP was 7.02 ± 1.30. For PMB1-PMB2 the average distance was 2.41 ± 0.64 mm, and for PMB2-PT it was 0.98 ± 0.33 mm.

**Table 2:** Prevalence MB2 canal in the mesiobuccal root of the maxillary second molars by gender and tooth position

	MB2		
	Absent	Present	Total
Female	167	105	272
	57,2%	40,7%	49,5%
Male	125	153	278
	42,8%	59,3%*	50,5%
Total	292	258	550
	100%	100%	100%
Rigth Side	151	127	278
	51,7%	49,2%	50,5%
Left Side	141	131	272
	48,3%	50,8%	49,5%
Total	292	258	550
	100%	100%	100%

* refers to *p* < 0.05

## Discussion

Despite their usefulness in locating the MB2 canal, magnification systems pose a series of limitations, such as a limited view of the clinical field, showing only superficially the mean orifice of the MB2 canal and not the entire root canal system. However, if access is not gained correctly, then magnification cannot provide an image of the area where the MB2 canal is located. In cases of inclined or rotated molars, magnification becomes less effective, since a severe to moderate angulation of the tooth prevents a good view of the pulpal floor. Stopko [8] stated that these microsurgical devices alone are insufficient to locate and instrument the MB2 canal in every case. On the other hand, conventional periapical x-rays are essential for the endodontic preoperative diagnosis and they are the most frequently used method for detecting accessory canals in everyday practice; nevertheless, the periapical x-ray can only provide two-dimensional information, which limits its diagnostic effectiveness. Furthermore, interpretation becomes difficult in terms of such factors as the superposition of anatomical structures, excessive bone density of the zygomatic arch or impacted teeth [13]. Barton et al. [16] and Abuabuara et al. [17] detected the MB2 canal in maxillary molars in 39.2% and 8% respectively through conventional periapical x-rays, demonstrating the low effectiveness of the method. Nattress & Martin [18] concluded that x-ray images were not reliable for detecting multiple canals. Therefore, it is very important to know and use additional tools to aid in detecting the MB2 canal in the diagnostic phase.

Patel et al. [13] reported CBCT as a non-invasive high-precision three-dimensional technique that increases the percentage of therapeutic success. Matherne et al. [19], using an in vitro human model, showed the superiority of CBCT over conventional x-rays in detecting the presence of accessory channels, and Blattner et al. [4], in an in vitro study, found CBCT to be a reliable method for the detection of the MB2 canal compared to the gold standard of physically sectioning the specimen.

Various studies have suggested the use of CBCT as an in vivo diagnostic method to detect the MB2 canal in maxillary molars [1, 3, 12, 14, 15]. The results obtained in this study revealed a prevalence of the MB2 canal in 69.81% in the 1MM, similar to that reported with the same diagnostic method by Kim et al. (63.59%) [20], Lee et al. (70.5%) [15], Betancourt et al. (68.75%) [12] and higher than the 52% reported by Zhang et al. [3] and the 8.78% by Zheng et al. [1]. The MB2 canal in the 2MM was identified in 46.90% of the cases, a percentage similar to the results reported using CBCT by Betancourt et al. (48%) [14], Lee et al. (42.2%) [15], and higher than the 34.32% by Silva et al. [21] and the 22% observed by Zhang et al. [3]. If two separate orifices blended into a single canal it was not considered to be a separate canal. This morphology is classified as Vertucci type 1 canal configuration and is the most seen in the second maxillary molars. This criteria is probably one of the reasons for the lower incidence of second mesiobuccal canals in this tooth.

The geometrical location of the MB2 canal has only been reported using in vitro studies [7, 22–24], however, a previous study by our group in second maxillary molars [14] demonstrated the efficiency of CBCT on MB2 canal location in vivo. This article is intended to expand the study sample used by Betancourt et al. [14], increasing the sample of 225 to 1,100 maxillary molars, also the study of the first maxillary molars were included. We observed that the MB2 canal was located in the 1MM 2.68 ± 0.49 mm palatally and 1.25 ± 0.34 mesially to the MB1 canal. In the 2MM it was located 2.41 ± 0.64 mm palatally and 0.98 ± 0.33 mm mesially, whereas Betancourt et al. [14], using the same technique, found it to be 2.2 ± 0.54 mm palatally and 0.98 ± 0.32 mesially to the MB1 canal. Gorduysus et al. [22] reported the MB2 location 1.65 ± 0.72 mm palatally and 0.69 ± 0.42 mesially to the MB1 canal in a combined study of first and second molars.

Our results regarding the location of the MB2 canal are lower than the results of Gilles & Reader [6], who located the MB2 canal mesially to the MB1 canal at a distance of 2.31 mm in the 1MM and 2.06 mm in the 2MM by scanning electronic microscopy, and Degerness & Bowles [24], who located it in the 1MM 1.2 ± 0.6 mm from the MB1 canal and in the 2MM 1.78 ± 0.6 mm through a stereomicroscope. Greater distances were reported by Kulid & Peter [25], who found no statistically significant differences between the 1MM and 2MM (1.82 ± 0.71mm), similarly to Görduysus et al. (1.81 ± 0.38 mm) [22]. This could be explained by the heightened sensitivity of in vitro studies or the use of microscopes with various magnifications that distort the images, whereas with CBCT the resolution of the resulting image is isotropic, i.e., the voxel, the minimum data unit, is equal in dimension on the 3 spatial axes, producing images without distortion or magnification (1:1).

We believe the variation in the geometric location of the MB2 canal mesially or palatally in relation to the MB1 canal depends on the type of study, because in vitro studies the anatomical relation and proportion on the arch is lost, where it is also not possible for all the axes and planes to be observed, which can be done with CBCT.

When relating the patient’s gender to the incidence of the MB2 canal, we obtained a statistically significant association. The 1MM (*p* = 0.005) and the 2MM (*p* = 0.000) was more frequent in men. These results are consistent with those reported by Fogel et al. [26] and Betancourt et al. [14]. However, Zheng et al. [1] and Betancourt et al. [12] found no difference. The smaller detection percentage of the MB2 canal in women could be explained by the demineralization and loss of bone mass in adults being three times greater in women [27], which would prevent the correct observation of the canal through computerized tomography due to lack of contrast.

The MB2 canal showed a high tendency to appear bilaterally, which is similar to that reported by Betancourt et al. [12], Betancourt et al. [14] and Lee et al. [15], all through in vivo CBCT images. This means that if a MB2 canal exists on one side, the clinician must consider searching in the contralateral mesiobuccal root.

Our results show that the observation of the MB2 canal is difficult at a higher age. This significant decrease in visibility through CBCT may be due to there being an increase in the porosity of the cortical bone and a reduction in bone mass after 50 years of age [28], which would cause an increase in the radiolucency of the bone and a subsequent lack of contrast with the MB2 canal, as this has a radiolucent structure. Another factor to consider is that with increasing age tertiary dentin dressing is being produced in certain places of the pulp-dentin interface due to the exposure of the tooth to external stressors, such as decay, dental trauma or restorative procedures. Finally, the elderly subjects presented greater canal calcification; therefore, the diameter of the additional canal is less than the diameter of the MB1 and palatal canals, a situation which is difficult to detect clearly on the CBCT images.

One significant problem which can affect the image quality and diagnostic accuracy of CBCT images is the scatter and beam hardening caused by high density neighboring structures, such as enamel, metal posts and restorations. If this scattering and beam hardening is associated close to or with the tooth being assessed the resulting CBCT images may be of minimal diagnostic value [29]. This difficulty did not arise in this study since the teeth with metallic restoration, intraradicular posts, root obturation or rehabilitated by means of a fixed prosthesis were excluded. Another point to consider is that the geometric location of the MB2 canal presents variations according to the height at which the measurements are taken; therefore, we recommend taking them at 1.0 mm (2 sections of 0.5mm) apically to the pulp chamber floor, because we regularly observed the MB2 at that level in every case where it was present.

These results indicate that CBCT is an effective, high-precision diagnostic tool for detecting and locating in vivo the MB2 canal in the mesiobuccal root of upper molars, thereby increasing the chances of endodontic success. This study demonstrates that the geometric location in vivo of the MB2 canal is possible through the methodology used in this article. Its tool helps to understand the root and canal morphology of maxillary molars in the diagnostic stage; as a result, it helps the clinician to perform the endodontic treatment safely, effectively, and predictively.

## Conclusion

The MB2 canal is found in 69.82% of the 1MM and 46.91% of the 2MM. In order to obtain the geometric location of the MB2 canal in the mesiobuccal root, we suggest using the center of the main mesiobuccal canal as a reference parameter and from there exploring 2.68 ± 0.49 mm in a palatal direction and 1.25 ± 0.34 mm in a mesial direction in the 1MM, while exploring 2.41 ± 0.64 mm in a palatal direction and 0.98 ± 0.33 mm in a mesial direction in the 2MM. Given our study results, we recommend that CBCT be considered a complementary diagnostic method before establishing an endodontic treatment in maxillary molars so as to obtain an optimal result.

## Abbreviations

1MM: First maxillary molar
2MM: Second maxillary molar
CBCT: Cone beam computed tomography
MB1: Mesiobuccal canal
MB2: Second mesiobuccal canal
MPR: Multiplanar reformatting
P: Palatal canal
PMB1: Central point of the mesiobuccal canal
PMB2: Central point of the MB2 canal
PP: Central point of the palatal canal
